# Mechanisms of Adsorption of Heavy Metal Cations from Waters by an Amino Bio-Based Resin Derived from Rosin

**DOI:** 10.3390/polym11060969

**Published:** 2019-06-03

**Authors:** Wanting Huang, Kaisheng Diao, Xuecai Tan, Fuhou Lei, Jianxin Jiang, Bernard A. Goodman, Yahong Ma, Shaogang Liu

**Affiliations:** 1 Guangxi Key Laboratory of Chemistry and Engineering of Forest Products, Guangxi Colleges and Universities Key Laboratory of Food Safety and Pharmaceutical Analytical Chemistry, School of Chemistry and Chemical Engineering, Guangxi University for Nationalities, Nanning 530008, China; hwt0506@126.com (W.H.); Diaokaisheng2010@163.com (K.D.); tanxc118@163.com (X.T.); leifuhou@163.com (F.L.); jiangjx@bjfu.edu.cn (J.J.); bernard_a_goodman@yahoo.com (B.A.G.); myh105060@163.com (Y.M.); 2 Department of Chemistry and Chemical Engineering, MOE Engineering Research Center of Forestry Biomass Materials and Bioenergy, Beijing Forestry University, Beijing 100083, China; 3 College of Physical Science and Engineering, Guangxi University, Nanning 530004, China

**Keywords:** aminated rosin-based resin, heavy metals, adsorption mechanism, density-functional-theory, interaction model

## Abstract

Rosin derived from conifer trees is used as the basis for a novel environmentally-friendly adsorbent prepared from a sustainable resource. After treatment with ethylenediamine, ethylenediamine rosin-based resin (EDAR) is produced, which possesses cation exchange capacity that is comparable to that of the best commercial synthetic resins. This is demonstrated by its application to the removal of Pb, Cd, and Cu from water, in single and multicomponent systems. Maximum uptake was obtained at pH 5 and in the order Pb(II) > Cd(II) > Cu(II). The maximum adsorption of Pb was ~1.8 mmol/g, but the adsorption process resembled the Freundlich isotherm, whereas the adsorption of Cd(II) and Cu(II) followed the Langmuir isotherm. In the multicomponent systems, there was direct competition between Pb and Cd for sorption sites, whereas the results with Cu indicated it had a preference for different types of sites compared to Pb and Cd. The EDAR resin could be efficiently regenerated and used repeatedly with only a small decrease in performance. Characterization of EDAR, and investigations of its adsorption mechanisms using physical, spectroscopic, and theoretical techniques, including fourier transform infrared spectroscopy (FTIR), ^13^C nuclear magnetic resonance (^13^C NMR), scanning electron microscope (SEM), Brunauer Emmett Teller (BET) method, elemental analysis, thermogravimetric analysis (TGA), and molecular dynamics calculations, showed that amino groups have a critical role in determining the cation adsorption properties. We conclude that this new adsorbent derived from an abundant natural material has the potential to make valuable contributions to the routine removal of heavy metal ions (HMs) from drinking water and wastewater.

## 1. Introduction

Water pollution by toxic heavy metal ions (HMs) is a cause of great global concern, because of their toxicity, non-biodegradability, and possible carcinogenicity [1,2]. Thus strict regulations and guidelines for the discharge of HMs have been established or recommended in many countries in response to public demands for clean water with extremely low levels of HMs. Considerable attention has been focused on the development of cost-effective procedures for HMs removal, and adsorption is generally considered to be an effective green separation technology, because of its simplicity, ability to reuse adsorbents, high selectivity, and high adsorption capacity. Numerous adsorbents, such as activated carbon, mesoporous silica, clay minerals, biosorbents, and polymeric resins have been used for the removal of HMs from water [3,4,5,6,7,8]. Among these adsorbents, synthetic polymers, particularly polyamine resins, have important advantages, which include high adsorption capacity, ready modification with functional groups, excellent stability, and easy regeneration [9]. Various chelating resins have been prepared by polymerization using cross-linkers, such as divinylbenzene, but these can then lead to secondary pollution if they subsequently break down [10,11], and in recent years more efforts have been paid to the development of adsorbents that are environmentally friendly, low-cost, and from renewable resources.

Rosin is an abundantly available natural product obtained from pine trees, which contains various isomerized acids (C_19_H_29_COOH > 90%) and some neutral components [12,13]. Its structure, based on phenanthrene, has excellent rigidity and improves resin structures when combined with synthetic polymers. Rosin-derived polymers have been synthesized for use in molecular separation and industrial synthesis [14]. Consequently, rosin has received increasing attention as a raw material for the synthesis of some new polymers with specific chemical structures and valuable properties [15,16]. However, despite great effort in developing applications using natural rosin and its derivatives, there is still very little literature on the use of rosin-based resins for the removal of HMs from drinking water and wastewaters.

For improved design of more effective adsorbents for the removal of HMs from water, it is important to understand the nature of their interactions. In the present work, products were characterized using various spectroscopic techniques, supported by theoretical calculations [17,18,19]. Quantum chemical calculations using the Density–Functional-Theory (DFT) provide an insight into interaction models, structures, and the binding abilities of various functional groups in the adsorbents towards HMs [20,21,22], and thus provide qualitative knowledge of adsorption mechanisms for the removal of HMs from water.

In this study, a novel ethylenediamine rosin-based resin (EDAR) was synthesized using free radical polymerization technology, and its performance evaluated for the removal of HM cations from aqueous solutions. Its effectiveness in removing Pb(II), Cd(II), and Cu(II) from water was assessed with single and multi-component systems, and compared with equivalent measurements with the initial rosin-based resin. Further insight into the adsorption behavior was obtained from thermodynamic and kinetic analyses of results from batch and fixed column systems.

## 2. Experimental Section

### 2.1. Materials

Natural rosin was collected from Wuzhou, Guangxi, China. Acrylic acid, maleic anhydride, glycol, methylacrylic acid, 2,2′-azoisobutyronitrile (AIBN), Pb(NO_3_)_2_, Cu(NO_3_)_2_·3H_2_O, Cd(NO_3_)_2_, MgCl_2_, HNO_3_, and Ca(NO_3_)_2_·were purchased from Sinopharm Chemical Reagent Co., Ltd, Shanghai, China. Synthetic humic acid (HA) was purchased from Aldrich Chemical Company, Milwaukee, WI, USA. All other chemicals were of analytical grade and all solutions were prepared with ultrapure water produced by a Milli-Q system (Advanatge A10, Millopore, Billerica, MA, USA). The main physiochemical properties of the commercial adsorbents used in this study are presented in Supporting Information (SI) Table S1.

### 2.2. Resin Synthesis and Characterization

**Scheme 1:**
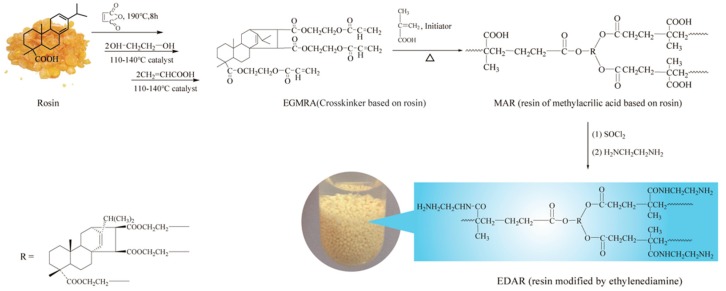
Illustration of the strategy of synthesis of aminated rosin-based resin (EDAR).

The cross-linking agent, ethylene glycol maleic rosinate acrylate (EGMRA) was synthesized via the Diels–Alder addition of natural rosin and maleic anhydride, and subsequent esterification with ethylene glycol and acrylic acid, according to our previous study [14]. EDAR was then synthesized as described in SI Text S1 and Scheme 1, where methylacrylic acid was used as the monomer, ethylene glycol maleic rosinate acrylate as the cross-linking reagent, peanut oil as the porogen, 2, 2′-azobisisobutyronitrile as the initiator, and ethylenediamine as the modifier. The final product was dried under vacuum at 50 °C for 8 h before characterization as described in SI Text S2, and used in absorption studies.

### 2.3. Batch Adsorption Studies

All experiments were performed at 25 °C in the dark and conducted in 150 mL conical flasks (containing 50 mL of solutions), which were shaken at 150 rpm for 24 h to ensure equilibrium. After a preliminary experiment to determine the optimum adsorbent dosage for high removal efficiency (SI Figure S1), batch experiments were employed to study the effects of various operating parameters, such as solution pH, ionic strength, co-existing ions, humic acid, and contact time, as well as the kinetics, isotherms, and thermodynamics of HMs adsorption. To investigate the adsorption performance of EDAR in natural waters, three samples from tap water, Yongjiang River, and Xiangsi Lake in Nanning were compared, and their characteristics are listed in SI Table S2. These water samples were filtered through a Millipore cellulose membrane filter (0.45 μm pore size), then their pH values were adjusted to 5.0, and finally spiked with 0.5 mM Pb(II), Cd(II), or Cu(II). All experiments were performed in duplicate.

### 2.4. Adsorption Dynamics, Regeneration and Adsorbent Stability

Solutions containing HMs were passed upwards through a glass column (Φ10 mm × 500 mm) packed with 1.2 g EDAR with a flow rate of 2.0 mL/min, and their concentrations at the column outlet were measured at various time intervals. Resin that had been used for adsorption of Pb(II) was regenerated with 0.1 M HCl solution, followed by 0.2 M NaHCO_3_ (for activation of the resin) at 25 °C, because of its weakly basic nature. The regenerated resin was washed with water until neutral pH prior to reuse. The adsorption–desorption cycle of Pb(II) was repeated 5 times to evaluate regeneration of the adsorption capacity.

### 2.5. Analysis Procedures

The concentrations of Pb(II), Cd(II), and Cu(II) in the residual solutions were determined by inductively coupled plasma atomic emission spectroscopy (ICP-AES) (Thermo, Franklin, MA, USA). Amounts of HMs adsorbed were calculated as the differences between the initial and residual concentrations (SI Text S3). Resin stability was assessed by shaking EDAR (100 mg) in water (200 mL) for 24 h at 30 °C and pH 6.0, then measuring the benzene series compounds (chlorobenzene, toluene, styrene, and xylene) and methacrylic acid using a gas chromatography-tandem mass spectrometer (GC-MS/MS, Bruker, Fremont, CA, USA).

### 2.6. Quantum Chemistry Calculations

All calculations were performed with the Gaussian 09 package (Gaussian, Inc., Wallingford, CT, USA) using dimers with different functional groups (e.g., methacryloyl, ethylenediamine or carboxylic) to simulate the donor moiety of the polymer chains [23]. All geometries were confirmed by frequency analysis that the ground state configuration and zero-point correction value were obtained. Various possible adsorption models of the complex system were explored and compared, but only those with the lowest energies are presented. Descriptions of the quantum chemistry calculations and the interaction energies (ΔE) are presented in detail in SI Text S4.

## 3. Results and Discussion

### 3.1. Characterization of EDAR

**Figure 1:**
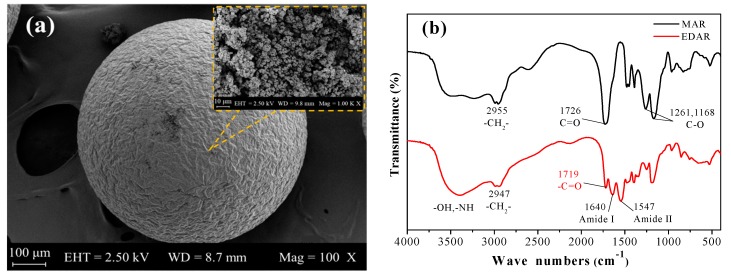

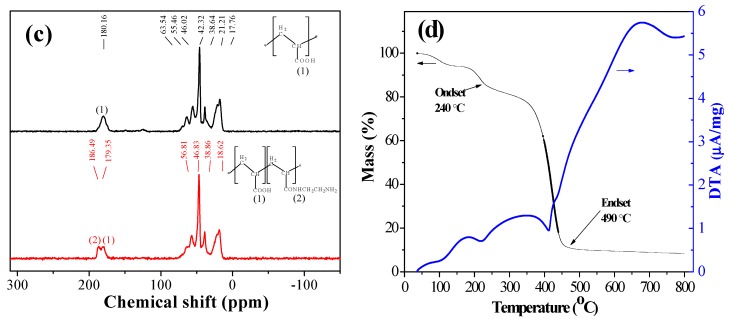
(**a**) SEM image of EDAR with different magnifications, (**b**) FTIR spectra of MAR and EDAR, (**c**) ^13^C NMR spectra of MAR and EDAR using CD_3_COOD/D_2_O as solvent, and (**d**) TGA curves for EDAR for the temperature range 40–800 °C.

The resins were characterized by FTIR, ^13^C NMR, SEM, BET, elemental analysis, and TGA. SEM images of EDAR showed that the EDAR samples are spherical beads (Figure 1a). In the IR spectra (Figure 1b), the characteristic peaks of amide I at 1640 cm^-1^ and amide II at 1547 cm^−1^ were present in EDAR but not in the resin (MAR) that was not treated with ethylenediamine. Some residual carboxylic acid groups characterized by peaks at around 3,500 cm^−1^ and 1,719 cm^−1^ remained in EDAR, which showed that only the H-bonded hydroxyl groups with a peak at ~3,250 cm^−1^ were modified by ethylenediamine. The ^13^C NMR spectra (Figure 1c) also confirmed the presence of an amide linkage at 186.5 ppm in EDAR along with a peak for the carbonyl carbon at ~180 ppm, which also occurred in MAR. Finally, elemental analyses showed that the N content of EDAR (5.41% by mass) was much higher than that of MAR (0.15% by mass), thus further confirming that amine groups were successfully grafted onto the linking units of MAR—it was calculated that 1 g EDAR contained 1.83 mmol amine groups. N_2_ adsorption–desorption isotherms for EDAR displayed a Type II profile according to the IUPAC classification with a specific area of 13.6 m^2^/g (SI Figure S2) [24]. The pore diameter and total pore volume were 6.44 nm and 0.017 cm^3^/g, respectively, and their distribution curve showed a concentration of diameters of ~50 nm. The TGA curve for EDAR (Figure 1d) showed that the resin was stable to 160 °C in air, which is adequate for use in water treatment, where elevated temperatures are not involved.

### 3.2. Comparison of Adsorption Capacities of EDAR and Various Commercial Adsorbents

The adsorption capacities of EDAR and MAR were compared with those of several commercial resins and granular activated carbon (GAC), which are currently used commercially for the removal of HMs from waters (SI Figure S3). The adsorption capacities of EDAR for Pb(II), Cd(II), and Cu(II) were much higher than those of MAR, which confirms that incorporation of aminated moieties greatly improves the HM adsorption properties, and thus implies that adsorption involves chelation and is not simply electrostatic attraction. Furthermore the adsorption capacities of EDAR for the HMs at pH 5.0 were similar to those of the resins D113 and IRC748, and higher than the resins DAX-8, IRA410, L-493, XAD-1180, XAD-4, and GAC. EDAR is a promising adsorbent for the removal of HMs from water, compared to the currently used petroleum-derived resins.

### 3.3. Single and Multi-Component Adsorption Isotherms

**Figure 2:**
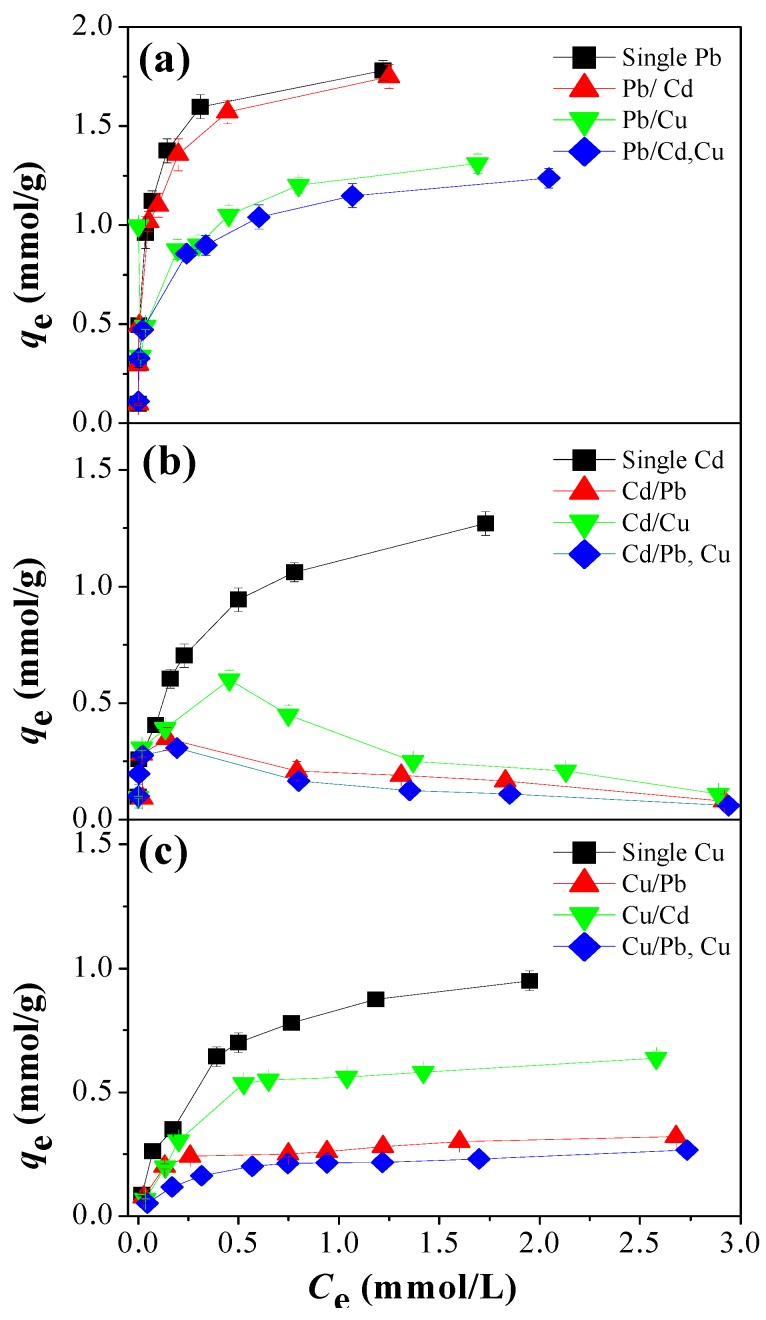
Adsorption isotherms for (**a**) Pb(II), (**b**) Cd(II), and (**c**) Cu(II) on EDAR in single, binary, and ternary systems. Experimental conditions: [HMs]o = 0.1, 0.3, 0.5, 0.8, 1.0, 1.5, 2.0, and 3.0 mM; [EDAR dosage] = 1.0 g/L; contact time = 24 h; pH 5.0; 25 °C.

The adsorption isotherms for Pb(II), Cd(II) and Cu(II) ions on EDAR in single, binary, and ternary systems are shown in Figure 2. It is noted that the amounts of adsorbed Cd(II) decreased with the increasing Pb(II) and Cu(II) concentrations above 0.1 mM in both binary and ternary systems. For single element systems, the experimental data were analyzed by the Langmuir and Freundlich isotherms (SI Text S5), and the parameters calculated from these models are listed in SI Table S3. Adsorption of Pb(II) is well described by the Freundlich model with high correlation coefficients (*R*^2^), whereas the adsorption of Cd(II) and Cu(II) were better fitted by the Langmuir model. This result suggests that some Pb(OH)_2_ species start to form on the surface of the adsorbent at this pH, whereas with Cd and Cu the adsorption of monolayers is consistent with their chelation to functional groups on the surface of the resin. The theoretical capacities for Pb(II), Cd(II), and Cu(II) on EDAR are about 1.8, 1.32, and 1.12 mmol/g, respectively, and are comparable to or greater than those of other bio-polymers in previous publications, which are listed in Supplementary Information Table S4.

Adsorption isotherms for HMs under competitive conditions are also shown in Figure 2. Uptake of the strongly adsorbed Pb(II) was unaffected by the presence of Cd(II), but compared to the single element was lowered by about 30% by the presence of an equivalent amount of Cu(II) in both binary and ternary systems. Furthermore, compared to the single element system, Cu(II) uptake was lowered by about 35% by the presence of an equivalent amount of Cd(II), and by ~65% by an equivalent amount of Pb(II) in both binary and ternary systems. The relationships for Cd(II), however, were more complex, and actually showed a decrease in the amounts adsorbed with increasing Cd(II) contents above 0.5 mmol/L when equivalent amounts of Pb(II) and/or Cu(II) were also present.

To analyze the nature of competition among Pb(II), Cd(II), and Cu(II), the Langmuir competitive isotherm (LCM) model [25], which describes adsorption of component i in the presence of component j (SI Text S6), was applied to the binary and ternary adsorption equilibrium data:

(1) $$q_{e,i}=q_{m,i}K_{L,i}C_{e,i}{\left(1+\sum_{j=1}^{n}K_{L,i}C_{e,j}\right)}^{-1}$$

where K_L,i_ is the individual Langmuir isotherm constant for each component. *q*_m,i_ and *C*_e,i_ are the maximum adsorption capacities and equilibrium concentrations in the mixture of solutes. The LCM model was able to fit the Pb(II) and Cu(II) adsorption data in the binary and ternary systems (*R*^2^ > 0.998), but not that of Cd(II) under competitive adsorption conditions (SI Figure S4).

### 3.4. Effect of Adsorption Parameters on HMs Adsorption

Various conditions for the adsorption of HMs on EDAR were also investigated, and are described in this section.

#### 3.4.1. pH

There was a general increase in adsorption with increasing solution pH up to pH 5 (SI Figure S5a), and the adsorption amounts for Pb(II), Cd(II), and Cu(II) containing 0.5 mmol/L were 0.49, 0.40, and 0.34 mmol/g, respectively. With each HM, the HM adsorption capacity of EDAR showed little change in the pH range 3.0–5.0, but there was an appreciable decrease when the pH was <3.0. The reason is successful competition between protons and HMs for the surface sites at highly acidic pH. There was no further increase in adsorption at pH values > 5.0, and with Cu(II) there was an appreciable decrease, presumably a consequence of hydrolysis reactions [26], which lead to precipitation of metal hydroxides at higher pH [18]. Thus, a pH of 5.0 was selected for all of the following adsorption experiments. The curves for the zeta potentials for both MAR and EDAR are presented as SI Figure S6. The isoelectric point (pH at which the net surface charge on the particle is zero) of MAR was approximately pH 4.7 whereas that of EDAR was nearly pH 10. Thus, although adsorption on MAR may be mainly electrostatic in nature, with EDAR electrostatic interactions are relatively minor, and complexation represents the main adsorption reaction.

#### 3.4.2. Salinity and Coexisting Compounds

Natural waters invariably contain various anions and cations that could potentially interfere with the adsorption of the HMs, either by competing with them for sites on the adsorbent, or competing with the adsorbent for the HM ions. Therefore, we also investigated the effects of Na(I), Mg(II), and Ca(II) cations, plus synthetic humic acid as a model anion, on the HM adsorption. Only slight decreases in adsorption of HMs were observed when NaCl concentrations were increased from 0 to 20 mM (SI Figure S5b), whilst increasing Mg and Ca in the 0 to 19 mM concentration range resulted in small progressive decreases in HMs adsorption (SI Figure S5c). Furthermore, there was little effect of HA at concentrations of 0 to 0.5 mg/L (SI Figure S5d).

#### 3.4.3. Adsorption Kinetics

Adsorption of HMs on EDAR showed an initial rapid increase, but then slowed as equilibrium was approached (after about 360 min) (SI Figure S5e and Text S8). The adsorption kinetics were fitted well with the pseudo-second-order model with high correlation coefficients (*R*^2^ > 0.99) (SI Table S5 and Figure S5f). The plots of *q*_t_ (mg/g) versus t^0.5^ for the intra-particle diffusion model were not linear (SI Figure S5g), although they could be separated into two linear regions. This suggests that there is an initial rapid step, followed by a slower step based on chelation, which involves some structural rearrangement.

#### 3.4.4. Temperature and Thermodynamics

Thermodynamic parameters (SI Text S7) for HMs adsorption on EDAR were obtained from experiments performed at three different temperatures (25, 35, and 45 °C) (SI Figure S5h). These show that the amount of adsorbed HMs increased with increasing temperature from 25 to 45 °C. Standard enthalpy change (Δ*H*º) and entropy change (Δ*S*º) were obtained by plotting ln *K*_d_ versus 1/T (SI Figure S5i). At all temperatures, the values of K_d_ were in the order Pb(II) > Cd(II) > Cu(II), which indicates that the affinity of EDAR resin for Pb(II) is higher than for Cd(II) or Cu(II). The negative values of Gibbs energy (Δ*G*º) (−1.73 to −17.77 kJ/mol) and positive values of Δ*H*º for all tested conditions and HMs (SI Table S6) indicate a spontaneous and endothermic adsorption process. The adsorption process is generally considered to involve chemical bonds if the absolute magnitude of Δ*H*º is >60 kJ/mol, and coordination exchange when Δ*H*º is about 40 kJ/mol, whereas for van der Waals and hydrophobic bonds it is ≤10 kJ/mol [27]. The absolute values of Δ*H*º for all the tested HMs were in the range 6.6 to 60 kJ/mol, suggesting that there is a combination of physisorption and covalent bonding. Furthermore, the positive values of Δ*S*º indicate that adsorption phenomenon for HMs on EDAR involves an associative mechanism, and that there is increased disorder at the solid-solution interface.

#### 3.4.5. Effect of Natural Water Matrix

We further investigated the potential of using EDAR for practical applications by determining its performance with natural waters spiked with Pb(II), Cd(II), or Cu(II) and adjusting the pH to 5.0. Results showed that the lake water had a significant effect on HMs adsorption because of its relatively high content of DOC (SI Figure S5j), but overall, these experimental results demonstrate the practical applicability of EDAR for removing HMs from environmental samples.

### 3.5. Dynamic Adsorption and Regeneration Studies

**Figure 3:**
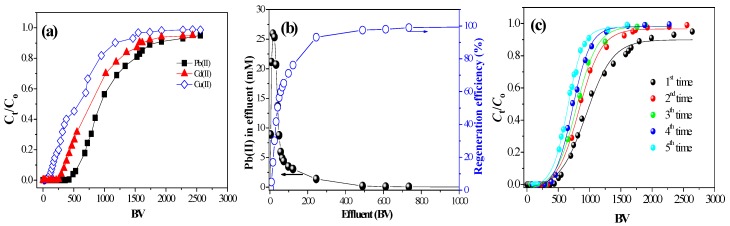
Dynamic adsorption and desorption profiles of HMs on EDAR at 25 °C. (**a**) Adsorption breakthrough curves (experimental conditions: [HMs] = 0.5 mM, flow rate was 2.0 mL/min at pH 5.0); (**b**) desorption of Pb(II) from EDAR column loaded with 1.63 mmol/g Pb using 0.1 mol/L HCl with a flow rate of 4 mL/min, and 7 h desorption time followed by 0.2 mol/L NaHCO_3_; and (**c**) breakthrough curves for Pb(II) on EDAR that had been subjected to successive regeneration treatments.

To further investigate its feasibility for practical use, we studied adsorption of Pb(II), Cd(II), and Cu(II) on fixed-bed columns of EDAR. The break-through curves (Figure 3a) indicate that the column performance was less satisfactory for Cu(II) than for Pb(II) or Cd(II) adsorption, and analysis of the behavior of the adsorbent–adsorbate system by the Thomas, Yoon–Nelson, Adams–Bohart models (SI Text S9) showed good fits to the dynamic adsorption data with the Thomas and Yoon–Nelson models with adsorption capacities of 1.64, 1.20, and 0.74 mmol/g for Pb(II), Cd(II), and Cu(II), respectively (SI Figure S7 and SI Table S7).

In order to examine its potential for reuse, the EDAR column that had been saturated with Pb(II) was regenerated with 0.1 M HCl solution followed by 0.2 M NaHCO_3_ at 25 °C. Most of the eluted Pb(II) was concentrated within about 50 bed volume (BV) (Figure 3b). When five consecutive adsorption–regeneration cycles were performed (Figure 3c), the dynamic adsorption capacity decreased progressively to 85.9%, 80%, 73.0%, and 67.5% of the initial value. In addition, the stability of the EDAR resin in the solution phase was assessed by monitoring the composition of the leachate, which did not detect styrene, chlorobenzene, xylene, or methacrylic acid.

### 3.6. Interaction Mechanisms and Models

#### 3.6.1. Spectroscopic Investigations

FTIR, X-ray photoelectron spectroscopy (XPS), and electron paramagnetic resonance (EPR) were used to investigate the adsorption interaction mechanisms of HMs with EDAR. The FTIR spectra of EDAR before and after adsorption of Pb(II), Cd(II), and Cu(II) at pH 5.0 are shown in SI Figure S8. The amide II (1549 cm^−1^) and N-H (3386 cm^−1^) peaks in EDAR shifted to higher frequencies in EDAR-Pb, EDAR-Cd, and EDAR-Cu, but the residual HO–C=O vibration (1716 cm^−1^) was not shifted. Thus the O=C-NH- and N-H groups of EDAR mainly participate in HMs adsorption [28,29].

**Figure 4:**
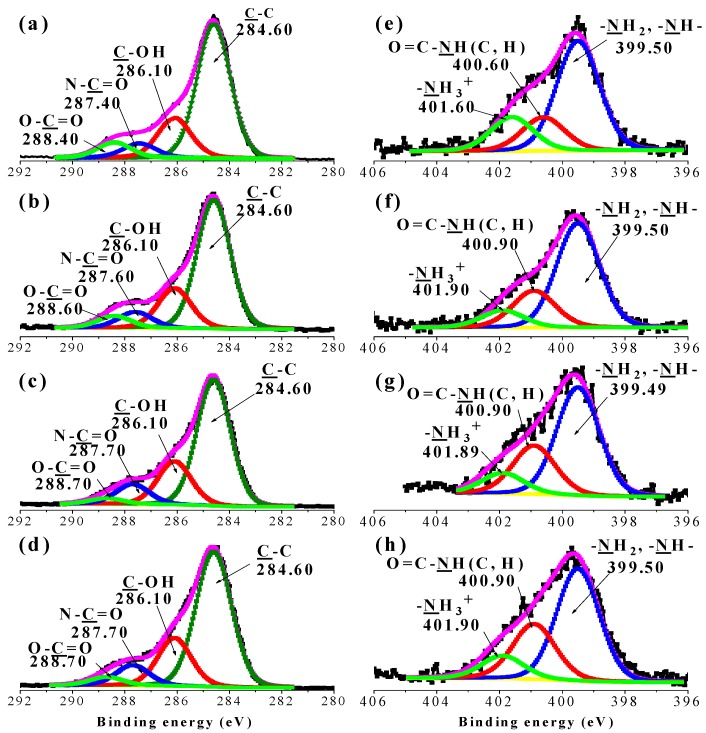
C1s (**a**–**d**) and N1s (**e**–**h**) X-ray photoelectron spectroscopy of EDAR and their deconvolutions into component peaks before (**a** and **e**), and after adsorption of Pb(II) (**b** and **f**), Cd(II) (**c** and **g**), and Cu(II) (**d** and **h**).

The XPS spectra of EDAR before and after HMs adsorption at pH 5.0 are presented in Figure 4 and SI Figure S9. The N1s spectra of EDAR were deconvoluted into three peaks at 399.50 eV, 400.41 eV, and 401.60 eV, corresponding to the N atom in R–NH_2_, O=C-N-H, and R-NH_3_^+^, respectively [5,30]. After adsorption of HMs, the energies of the N1s peaks of R-NH_3_^+^ and O=C-NH_2_^-^ increased by 0.3 eV, whereas those of R–NH_2_ were essentially unchanged (Figure 4e–h). This result suggests that HMs bind to EDAR by displacing H^+^ from R-NH_3_^+^ and not to the lone-pair of electrons at R–NH_2_ (Figure 4e–h). Deconvolution of the C1s spectra of EDAR produced four peaks with binding energy of 284.60, 286.10, 287.40, and 288.40 eV, which can be assigned to C atoms in the forms of C-C, C-O (alcoholic), N-C=O (amide), and O-C=O (carboxylate), respectively [31,32]. After HMs adsorption, the C1s binding energy of -C=O peaks increased by about 0.2 eV (Figure 4a–d). The O1s spectra were deconvoluted into two component peaks at 531.39 eV and 532.89 eV, which are mainly associated with the –C=O and C-O groups. After HM adsorption, the O1s binging energy of the –C=O peaks decreased by about 0.1 eV (SI Figure S9), which is consistent with a decrease in electron density on the O atoms [31]. Consequently, both FTIR and XPS spectra suggest that both N and O (C=O) atoms are involved in the bonding between HMs and EDAR.

**Figure 5:**
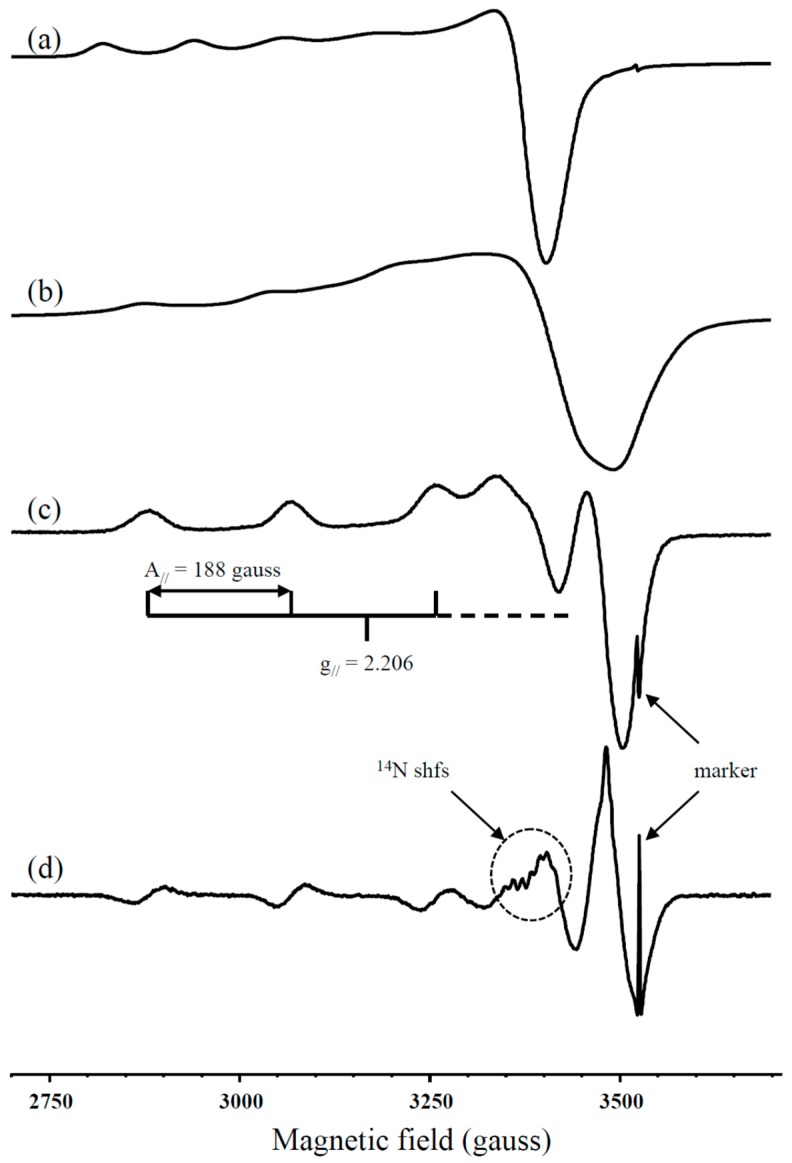
1^st^ derivative EPR spectra at room temperature (~25 °C) of Cu(II) adsorbed on (**a**) MAR, and (**b**) EDAR. Spectra from lower adsorption levels of Cu(II) on EDAR are also shown as (**c**) 1^st^ derivative, and (**d**) 2nd derivative recordings.

Since Cu(II) is paramagnetic, EPR spectroscopy was used to further investigate the coordination environment of Cu(II) adsorbed on MAR and EDAR at pH 5.0. The spectrum from the MAR sample (Figure 5a) resembles that of the hydrated Cu(II) ion, whereas the spectrum of Cu(II)-saturated EDAR (Figure 5b) corresponds to copper in complexes with tetragonal symmetry. This spectrum is broadened by dipolar interactions between neighboring Cu(II) ions, which makes it difficult to determine the spectral parameters accurately, but adsorbing a lower amount of Cu gave a better resolved spectrum (Figure 5c). A 2^nd^ derivative recording of this sample showed the presence of some ^14^N superhyperfine structure (shfs) (Figure 5d), which is similar to that previously reported for a N-rich sample of EDAR [33]. This shfs is not sufficiently well resolved for direct determination of the number of nitrogen atoms bound to the copper, but Liu et al [33] reported that the g_//_- and A_//_(Cu)-values for copper adsorbed on a similar resin sample are consistent with the copper being coordinated to four nitrogen atoms.

#### 3.6.2. Interaction Models

**Figure 6:**
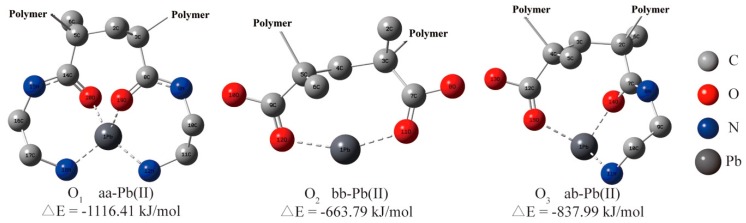
Dimeric structures used to simulate potential basic units of EDAR polymers for coordination with Pb(II) (for clarity, hydrogen atoms are omitted from all structures).

The possible modes of coordination of HMs to EDAR was further investigated using quantum chemical calculations with Pb(II) as the target metal. These calculations used three dimeric structures as models, namely complete amination (aa), partial amination (ab), and non-amination (bb). It should be noted that steric effects in the polymer on adsorption of HMs was not considered and the focus was only on the interaction between the dimer structure of EDAR and Pb. Optimized structures of possible EDAR-Pb(II) complexes are shown in Figure 6 and SI Figure S10, in which H1–H3 are the hypothetical initial structures of aa-Pb(II), H4–H6 are those for ab-Pb(II), and H7 is that for bb-Pb(II), and O_1_, O_2_, O_3_ are the optimized structures for aa-Pb(II), ab-Pb(II), bb-Pb(II), respectively.

For Pb(II) adsorption, the ΔE values for the models aa-Pb(II), ab-Pb(II) and bb-Pb(II) were −1116.41, −837.99, and −663.79 kJ/mol, respectively, thus showing a preference of Pb(II) for N compared to O coordination. Although the bonding to the actual resin is complicated by other factors, such as the steric arrangements of the functional groups, these results strongly support the experimental observation that amination of MAR can significantly improve its adsorption capacity for HMs. Normally, bond length in functional groups will lengthen when coordinated to metals, and the lengths of bonds coordinated to Pb, namely C8 = O19, C11-N12, C14 = O28, and C17-N18 were increased by 0.042 Å, 0.045 Å, 0.037 Å, and 0.029 Å, respectively (SI Table S8). These results also confirm the quadridentate configuration of Pb(II) adsorbed on EDAR, and thus its resemblance to the Cu(II) coordination revealed by the EPR measurements.

## 4. Conclusions

The novel resin EDAR with aminated functional groups exhibited excellent adsorption properties for the HM ions Pb(II), Cd(II), and Cu(II), and compared favorably with many commercial resins. Adsorption was pH-dependent and most effective for the range 4.0–6.0. The adsorption capacity was in the order Pb(II) > Cd(II) > Cu(II) in single, and competitive systems. Apart from Cd–Cu and Cd–Pb–Cu systems, the multicomponent systems were fitted well by the Langmuir competitive model. Various spectroscopic investigations showed that both N and O atoms in EDAR are involved in the coordination of Pb(II), Cd(II), and Cu(II), whereas quantum chemical calculations indicated that coordination to the amine groups was energetically the most favorable arrangement. Overall, the combination of thermodynamic and spectroscopic results with quantum mechanics calculations indicates that both chemical bonding and electrostatic processes are involved in HM adsorption on EDAR. In addition, the HMs could be successfully removed from the EDAR with 0.1 M HCl, and the resin regenerated for further use by treating with NaHCO_3_ solutions. Finally, EDAR was applied successfully to the removal of HMs from natural waters, and further studies to validate its performance in various practical applications are currently underway. Consequently, we believe that the rosin-based resin has great potential to replace of the petroleum-derived resins that are currently used.

## Supplementary Materials

The following are available online at https://www.mdpi.com/2073-4360/11/6/969/s1, Figure S1. Effect of adsorbent dosage on HMs adsorption on EDAR (a) and removal from solution (b). Experimental conditions: [HMs]o = 0.5 mM, contact time = 24 h, pH 5.0, 25 °C. Figure S2. N2 adsorption-desorption isotherms for EDAR. Figure S3. Adsorption of 0.5 mM Pb(II), Cd(II) and Cu(II) on various adsorbents at pH 5.0 and 25 °C using 1.0 g/L adsorbent dosage. Figure S4. Experimental results for the competitive adsorption of Pb, Cd, and Cu in binary systems presented in the linear form of the Langmuir competitive model. Figure S5. Effect of different parameters on the adsorption of Pb(II), Cd(II) and Cu(II) by EDAR. (a) solution pH; (b) ionic strength; (c) Ca(II) and Mg(II); (d) HA. (e) contact time; (f) Pseudo-second-order; (g) Intra-particle diffusion model; (h) temperature; (i) Plots of ln kd versus 1/T for the adsorption of Pb(II), Cd(II) and Cu(II) by EDAR; (j) different water matrixes. Experimental conditions: [HMs] = 0.5 mM (except for adsorption isotherm test), [EDAR dosage] = 1.0 g/L, pH 5.0(except for pH test), 25 °C (except for temperature test). Figure S6. Variation in zeta potential of EDAR and MAR as a function of pH. Figure S7. Comparison of experimental curves for adsorption of Pb(II), Cd(II), and Cu(II) on EDAR with predicted breakthrough curves obtained from the Thomas, Adams Bohart, and Yoon–Nelson models. Figure S8. FTIR spectra of EDAR before and after adsorption of Pb(II), Cd(II), and Cu(II). Figure S9. XPS O1s spectra of EDAR before (a) and after adsorption of Pb(II) (b), Cd(II) (c), and Cu(II) (d). Figure S10. Initial geometries (H1-H7) used for calculations of Pb(II) coordination to EDAR, and the corresponding optimized coordination geometries (O1-O3). Table S1. Physicochemical properties of the adsorbents used in the study. Table S2. Main characteristics of the natural water samples used in this study. Table S3. Adsorption isotherm model constants for single systems at 25 °C. Table S4. Comparison of adsorption capacities of various adsorbents for HMs at pH 5.0. Table S5. Kinetic parameters for the adsorption of Pb(II), Cd(II), and Cu(II) on EDAR. Table S6. Thermodynamic parameters for the adsorption of HMs on EDAR (0.5 mM HMs). Table S7. Parameters for the Thomas, Adams–Bohart, and Yoon–Nelson dynamic adsorption models fitted for Pb(II), Cd(II), and Cu(II). Table S8. Changes in lengths of selected bonds in EDAR model aa as a result of complexation with Pb(II) (in A°).
