# Supplementary material for: Mechanisms of Adsorption of Heavy Metal Cations from Waters by an Amino Bio-Based Resin Derived from Rosin
Source: Polymers (Basel). 2019 Jun 3;11(6):969. doi: 10.3390/polym11060969 (PMC6630295; doi:10.3390/polym11060969)
Supplement: Supplementary file 1 [file polymers-11-00969-s001.pdf]

Supporting information

Mechanisms of adsorption of heavy metal cations from waters by an amino bio-based resin derived from rosin

Wanting Huang[1], Kaisheng Diao[1], Xuecai Tan[1], Fuhou Lei[1], Jianxin Jiang[1, 2], Bernard A. Goodman[1, 3], Yahong Ma[1], Shaogang Liu[1 *]

[1] *Guangxi Key Laboratory of Chemistry and Engineering of Forest Products, Guangxi Colleges and Universities Key Laboratory of Food Safety and Pharmaceutical Analytical Chemistry, School of Chemistry and Chemical Engineering, Guangxi University for Nationalities, Nanning 530008, Guangxi, China.*

[2] *Department of Chemistry and Chemical Engineering, MOE Engineering Research Center of Forestry Biomass Materials and Bioenergy, Beijing Forestry University, Beijing 100083, China.*

[3] *College of Physical Science and Engineering, Guangxi University, Nanning 530004, Guangxi, China.*

Corresponding author: (S G, Liu) Phone: 86-0771-3267019; e-mail: liushaogang2005@163.com

The supporting information includes 31 pages, 9 texts, 9 figures, and 7 tables.

Contents

Text S1. Resin synthesis.

Text S2. Characterization of resin.

Text S3. Calculation of adsorption amount and removal efficiency of HMs.

Text S4. Quantum chemical calculation.

Text S5. Adsorption isotherm models.

Text S6. Adsorption isotherm models for multi-component systems.

Text S7. Calculation of thermodynamic parameters.

Text S8. Equations for kinetic models.

Text S9. Dynamic adsorption performance models.

Fig. S1. Effect of adsorbent dosage on HMs adsorption on EDAR (a) and removal from solution (b). Experimental conditions: $[HMs]_o$ = 0.5 mM, contact time = 24 h, pH 5.0, 25 °C.

Fig. S2. N_2 adsorption-desorption isotherms for EDAR.

Fig. S3. Adsorption of 0.5 mM Pb(II), Cd(II) and Cu(II) on various adsorbents at pH 5.0 and 25°C using 1.0 g/L adsorbent dosage.

Fig. S4. Experimental results for the competitive adsorption of Pb, Cd, and Cu in binary systems presented in the linear form of the Langmuir competitive model.

Fig. S5. Effect of different parameters on the adsorption of Pb(II), Cd(II) and Cu(II) by EDAR. (a) solution pH; (b) ionic strength; (c) Ca(II) and Mg(II); (d) HA. (e) contact time; (f) Pseudo-second-order; (g) Intra-particle diffusion model; (h) temperature; (i) Plots of ln k_d versus 1/T for the adsorption of Pb(II), Cd(II) and Cu(II) by EDAR; (j) different water matrixes. Experimental conditions: [HMs] = 0.5 mM (except for adsorption isotherm test), [EDAR dosage] = 1.0 g/L, pH 5.0(except for pH test), 25°C (except for temperature test).

Fig. S6. Variation in zeta potential of EDAR and MAR as a function of pH.

Fig. S7. Comparison of experimental curves for adsorption of Pb(II), Cd(II), and Cu(II) on EDAR with predicted breakthrough curves obtained from the Thomas,

Adams Bohart, and Yoon–Nelson models.

Fig. S8. FTIR spectra of EDAR before and after adsorption of Pb(II), Cd(II), and Cu(II).

Fig. S9. XPS O1s spectra of EDAR before (a) and after adsorption of Pb(II) (b), Cd(II) (c), and Cu(II) (d).

Fig. S10. Initial geometries (H1-H7) used for calculations of Pb(II) coordination to EDAR, and the corresponding optimized coordination geometries (O1-O3).

Table S1. Physicochemical properties of the adsorbents used in the study.

Table S2. Main characteristics of the natural water samples used in this study.

Table S3. Adsorption isotherm model constants for single systems at 25 °C.

Table S4. Comparison of adsorption capacities of various adsorbents for HMs at pH 5.0.

Table S5. Kinetic parameters for the adsorption of Pb(II), Cd(II), and Cu(II) on EDAR.

Table S6. Thermodynamic parameters for the adsorption of HMs on EDAR (0.5 mM HMs).

Table S7. Parameters for the Thomas, Adams–Bohart, and Yoon–Nelson dynamic adsorption models fitted for Pb(II), Cd(II), and Cu(II).

Table S8. Changes in lengths of selected bonds in EDAR model aa as a result of complexation with Pb(II) (in A°).

Supplementary References

Text S1. Resin Synthesis.

Polymerization was performed in a mixture of monomer (9.5 g methylacrylic acid), crosslinking agent (1.0 g EGMRA, prepared as described in Scheme S1)), initiator (0.2 g AIBN), and toluene (50 mL) at 85 °C for 3 h, whilst being deoxygenated with nitrogen gas. The rosin-based resin of methylacrylic acid (MAR) was separated by filtration, and washed repeatedly with deionized water (60 °C) and ethanol to remove residual materials; the yield was 73%. The synthetic process is shown in Scheme 1.

EDAR was then synthesized by adding ethylenediamine dropwise to refluxing MAR (10 g) in thionyl chloride (50 mL) and the mixture refluxed for 4 h; prior to the reaction, MAR was soaked in benzene (50 mL) at room temperature for 12 h, EDAR was filtered, washed with deionized water, then subsequently extracted with ethanol for 4 h. The final product was dried under vacuum at 50 °C for 8 h before characterization and use in absorption studies. The overall synthetic process is shown in Scheme 1.

Text S2. Characterization of Resin.

^{13}C nuclear magnetic resonance (NMR) spectra were obtained at room temperature (～22 °C) in 1% deuterated acetic acid using a Bruker AMX 500 spectrometer (100.62 MHz for ^{13}C NMR). Elemental analyses were performed with an Elemental Analyzer (EA, Elemental Vario MICRO) and Fourier transform infrared (FT-IR) spectra were obtained with a Nicolet 5700 FTIR spectrometer (Thermo Nicolet Co., USA). Sample morphology was examined by field emission scanning electron microscopy (FE-SEM, SUPRA 55, Carl Zeiss AG), and thermal stability was tested using a TGA Q50 simultaneous thermal analyzer (Waters, USA), in which the sample was heated from 35 to 700 °C at a rate of 10 °C/min under a N_2 flow rate of 100 mL/min. Zeta potentials of EDAR and MAR were measured in a Zetasizer 2000 Analyzer (Malvern, Mastersizer 2000 Instruments Co., USA). The BET specific surface areas and pore sizes of adsorbents were determined by N_2 adsorption–desorption isotherms using an automatic surface analyzer (ASAP2020, Micromeritics, USA). Chemical analyses of EDAR and its HM-loaded composites were conducted by X-ray photoelectron spectroscopy (XPS, ESCALAB 250), in which the XPSPEAK41 software was used to fit the XPS spectra. Electron paramagnetic resonance (EPR) spectra were acquired as either 1st or 2nd derivatives of the microwave absorption at room temperature from selected samples (~25 °C) on a Bruker A300 X-band spectrometer equipped with a Gunn diode microwave source and a high sensitivity resonance cavity. Spectral acquisition parameters were: 5 mW microwave power, 100 kHz modulation frequency, 5 gauss modulation amplitude, center field 3100 gauss, scan range 1500 gauss, and resolution 2048 points.

Text S3. Calculation of adsorption amount and removal efficiency of HMs

The amounts of HMs adsorbed by EDAR and their removal efficiency from water were calculated using the following equations:

$$q_t = \frac{(C_0 - C_t)V}{m} \tag{S1}$$

$$\text{HM removal efficiency } \% = \frac{(C_0 - C_t)}{C_0} \times 100 \tag{S2}$$

where C_0 and C_t (mmol/L) are the concentrations of HMs in aqueous solution initially and at time t, respectively; q_t (mmol/g) is the amount of HMs adsorbed at equilibrium, v is the volume of HMs solution (L), m is the mass of adsorbent (g).

Text S4. Quantum chemical calculations.

These models and metal complexes were preliminarily optimized by Molecular Mechanics (MM+) prior to more accurate calculation. The geometries of all species were fully optimized by density functional theory (DFT) without restrictions, using the Becke3 parameter exchange function of the Lee-Yang-Parr correlation function (B3LYP) [1] with the 6-31G ** basis set for the C, H, O, N atoms except that the metal ions were in the pseudopotential basis set of Lanl2dz. Single point frequency calculations of these optimized geometries ensured their minimum energy structures. The interaction energy (ΔE) between adsorbate and absorbent can evaluate the relative electron donating ability of dimers with different functional groups, and its use is feasible for describing the complexation of a given metallic ion. It is defined by Eq. (2).

$$\Delta E = E(DM) - [E(M) + E(D)] \qquad (S3)$$

where E(DM) is the total energy of the complex, E(M) is the acceptor energy of free metal ions, and E(D) the donor energy of free adsorbent dimers.

Text S5. Adsorption isotherm models

To quantify the adsorption capacity of EDAR, isotherms for adsorption of Pb(II), Cd(II), and Cu(II) on EDAR at 25 °C were investigated by the Langmuir and Freundlich models using the following equations:

Langmuir model [2]: $q_e = \frac{q_m K_L C_e}{1 + K_L C_e}$ (S4)

Freundlich model [3]: $q_e = K_F C_e^{\frac{1}{n}}$ (S5)

where C_e is the equilibrium concentration of the metal ion (mM), q_e is the equilibrium adsorption capacity (mmol/g), q_m (mmol/g) and K_L (L/mmol) are the maximum adsorption capacity and Langmuir constant, respectively. K_F [(mmol/g) $(mmol/L)^{1/n}$] and n are the Freundlich constants related to adsorption capacity and adsorption intensity parameter, respectively.

Text S6. Adsorption isotherm models for multi-component systems

For binary and ternary systems [4],

$$\frac{C_{e,1}}{C_{e,2}\,q_{e,1}} = \frac{C_{e,1}}{q_{m,1}\,C_{e,2}} + \frac{K_{L,2}}{K_{L,1}\,q_{e,1}} \qquad \text{(S6)}$$

$$\frac{C_{e,1}}{q_{e,1}(K_{L,2}C_{e,2} + K_{L,3}C_{e,3})} = \frac{C_{e,1}}{(K_{L,2}C_{e,2} + K_{L,3}C_{e,3})q_{m,1}} + \frac{1}{q_{m,1}K_{L,1}} \qquad \text{(S7)}$$

where plots of $C_{e,1}/C_{e,2}q_{e,1}$ as a function of $C_{e,1}/C_{e,2}$, and $C_{e,1}/q_{e,1}(K_{L,2}\ C_{e,2} + K_{L,3}\ C_{e,3})$ as a function of $C_{e,1}/\ (K_{L,2}\ C_{e,2} + K_{L,3}\ C_{e,3})$ give intercepts of $K_{L,2}/\ K_{L,1}q_{e,1}$ and $1/q_mK_{L,1}$ for the binary and ternary systems, respectively, with slopes of $1/q_{m,1}$ in each case.

Text S7. Calculation of thermodynamic parameters

Thermodynamic parameters, such as the standard Gibbs energy change ($\Delta G°$), enthalpy change ($\Delta H°$), and entropy change ($\Delta S°$) for the adsorption of HMs on EDAR, which are calculated by eqns. S8–S10, can provide in-depth information about the energetic changes associated with adsorption. The equations can be written as follows:

$$\Delta G^{\mathrm{o}} = -RT \ln \mathrm{K_d} \quad \text{(S8)}$$

$$\Delta G^{\mathrm{o}} = \Delta H^{\mathrm{o}} - T\Delta S^{\mathrm{o}} \quad \text{(S9)}$$

$$\ln \mathrm{K_d} = \frac{\Delta S^{\mathrm{o}}}{R} - \frac{\Delta H^{\mathrm{o}}}{RT} \quad \text{(S10)}$$

where the distribution coefficient (K_d = C_{ad}/C_e) is a dimensionless parameter, and represents the ratio of the concentration of solute adsorbed on the EDAR (C_{ad}) to the residual concentration of the solute in solution at equilibrium (C_e).. R is the universal gas constant (8.314 J/mol/K), and T is the absolute temperature. Standard enthalpy change ($\Delta H°$) and entropy change ($\Delta S°$) were obtained by plotting of ln K_d versus 1/T (SI Figure S5). At all temperatures, the values of K_d were in the order Pb(II) > Cd(II) > Cu(II), which indicates that the affinity of EDAR resin for Pb(II) (a measure of adsorption ability of EDAR for HMs), is higher than for Cd(II) or Cu(II).

Text S8. Equations for kinetic models

Understanding the reaction kinetics is important for improving the design of adsorption systems. These can be characterized by pseudo-first-order, pseudo-second-order, and intra-particle diffusion kinetic models, which can be written as follows:

First-order kinetic equation [5]: $\lg(q_e - q_t) = \lg q_e - \frac{k_1}{2.303}t$ (S11)

Second-order kinetic equation [6]: $\frac{t}{q_t} = \frac{1}{k_2 q_e^2} + \frac{t}{q_e}$ (S12)

Intra-particle diffusion equation [7]: $q_t = k_p t^{0.5} + C$ (S13)

where q_e and q_t are the amounts (mmol) of adsorbate adsorbed per gram of adsorbent at equilibrium and at time t (min) respectively; k_1 (1/min) and k_2 (g/mmol/min) are the adsorption rate constants of the pseudo-first-order and pseudo-second-order kinetics reactions, respectively; k_{id} is the intraparticle diffusion rate constant (mmol/g/min$^{0.5}$); and c_i is a constant associated with the boundary layer thickness.

Text S9. Dynamic adsorption performance models.

The Thomas, Yoon-Nelson, and Adams-Bohart models can be used to predict column adsorption performance. The relevant equations are written as follows (Eqs. S14-S16):

Thomas model [8]:

$$\frac{C_t}{C_0} = \frac{1}{1+\exp(K_{\mathrm{Th}} q_0 m / Q - K_{\mathrm{Th}} C_0 t)} \qquad \text{(S14)}$$

Yoon-Nelson model [9]:

$$\frac{C_{\mathrm{t}}}{C_0} = \frac{\exp(K_{\mathrm{YN}} t - \tau K_{\mathrm{YN}})}{1+\exp(K_{\mathrm{YN}} t - \tau K_{\mathrm{YN}})} \qquad \text{(S15)}$$

Adams-Bohart model [10]:

$$\frac{C_{\mathrm{t}}}{C_0} = \exp\left(k_{\mathrm{AB}} C_0 t - k_{\mathrm{AB}} N_0 \frac{Z}{F}\right) \qquad \text{(S16)}$$

where k_{Th} is the Thomas rate constant (L/min/mmol); q_0 is the uptake of HMs per g of the adsorbent (mmol/g) at equilibrium; m is the amount of adsorbent in the column (g); V_{eff} is the effluent volume (mL); C_0 and C_t are the initial HM concentrations and at time t (mM); v is flow rate (L/min). The value of t is time (min, t = V_{eff}/V). k_{AB} is the kinetic constant (ml/mmol/min), F is the linear velocity calculated by dividing the flow rate by the column section area (cm/min), Z is the bed depth of column and N_0 is the saturation concentration (mM). k_{YN} is the rate constant (1/min) and is τ, the time required for 50% adsorbate breakthrough (min).

Fig. S1

The effect of adsorbent dose on the adsorption process was studied by varying the dose of EDAR in the range 0.2–2.0 g/L. As shown in Figure S1, the removal efficiencies for HMs increased with adsorbent dose for low adsorbent concentration, but reached a maximum with Pb for 0.5 g/L, although with both Cd and Cu there was increased adsorption up to 2.0 g/L. Therefore, taking into account the efficiency and economy of operation, 1.0 g/L (solid-to-liquid ratio) was chosen as the optimum adsorbent dosage for all subsequent experiments.

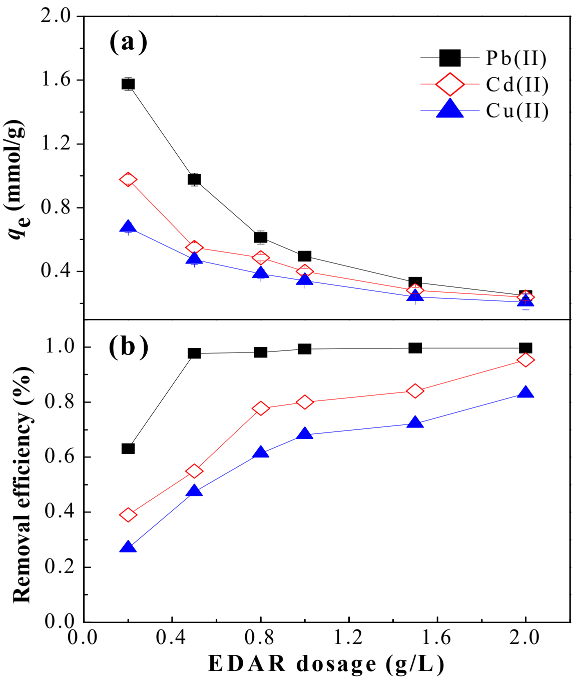

Fig. S1. Effect of adsorbent dose on HMs adsorption on EDAR (a) and removal from solution (b). Experimental conditions: $[HMs]_o$ = 0.5 mM, contact time = 24 h, pH 5.0, 21 ± 1 °C.

Fig. S2

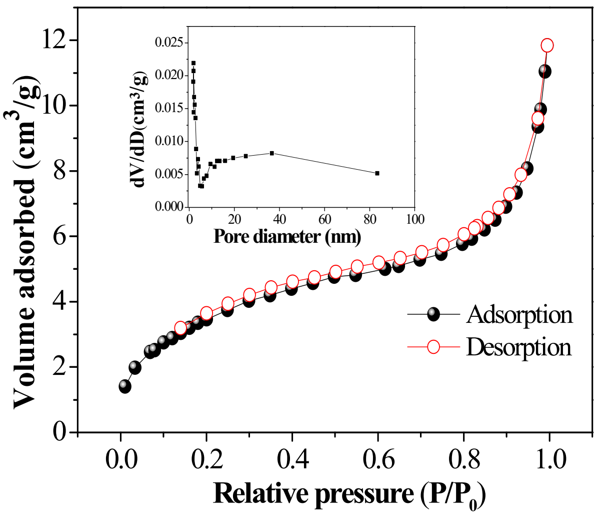

Fig. S2. N_2 adsorption-desorption isotherms for EDAR.

Fig. S3

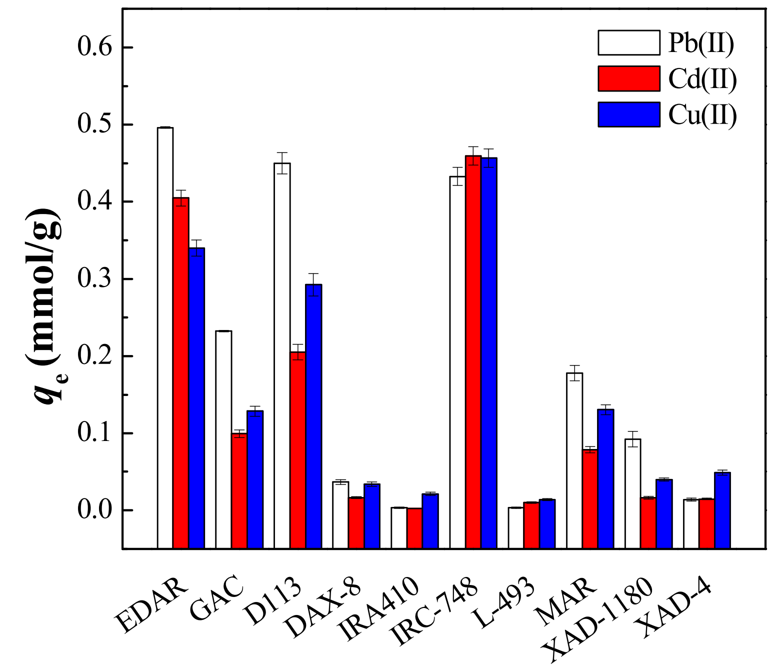

Fig. S3. Adsorption of 0.5 mM Pb(II), Cd(II) and Cu(II) on various adsorbents at pH 5.0 and 25 °C using 1.0 g/L adsorbent.

Fig. S4

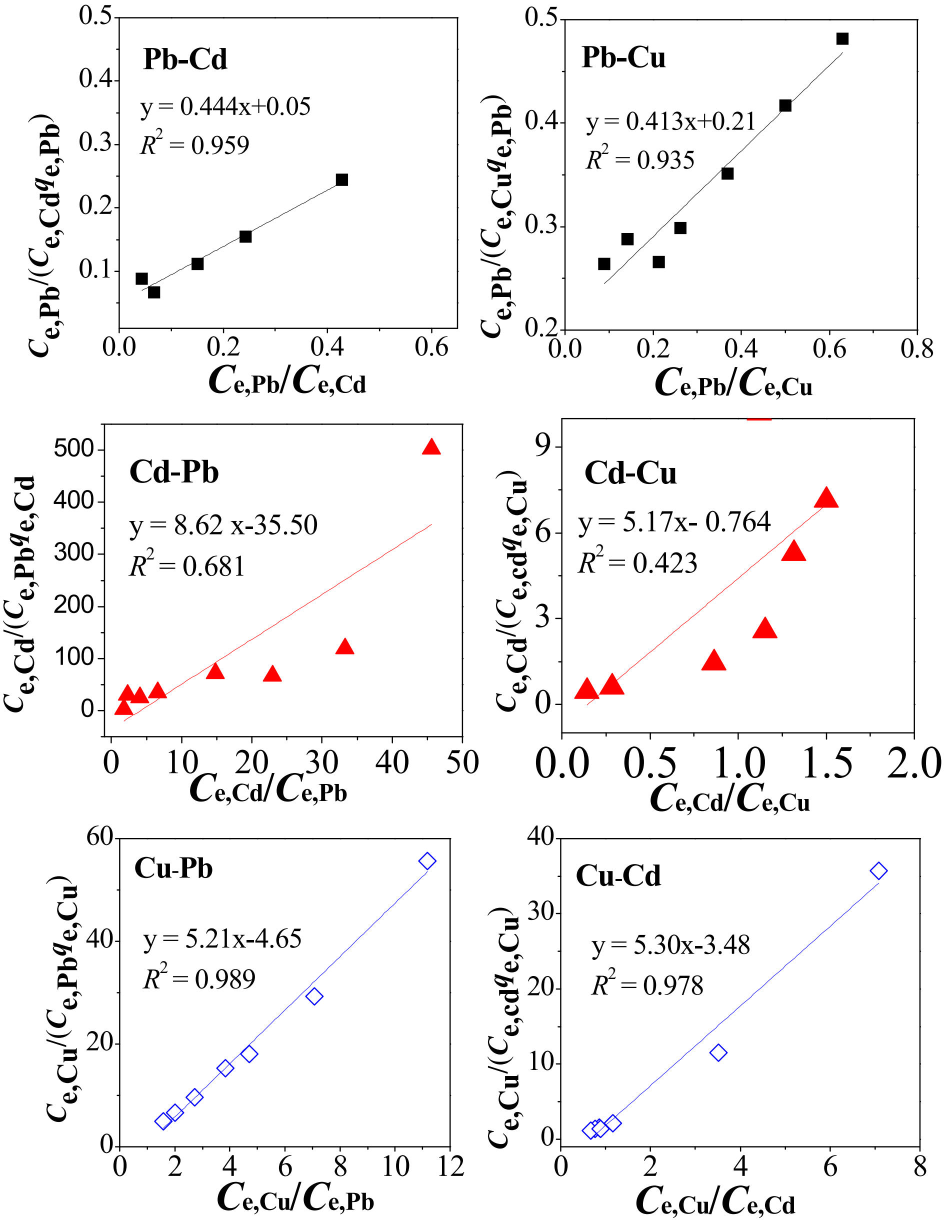

Fig. S4. Experimental results for the competitive adsorption of Pb, Cd, and Cu in binary systems presented in the linear form of the Langmuir competitive model.

Fig. S5

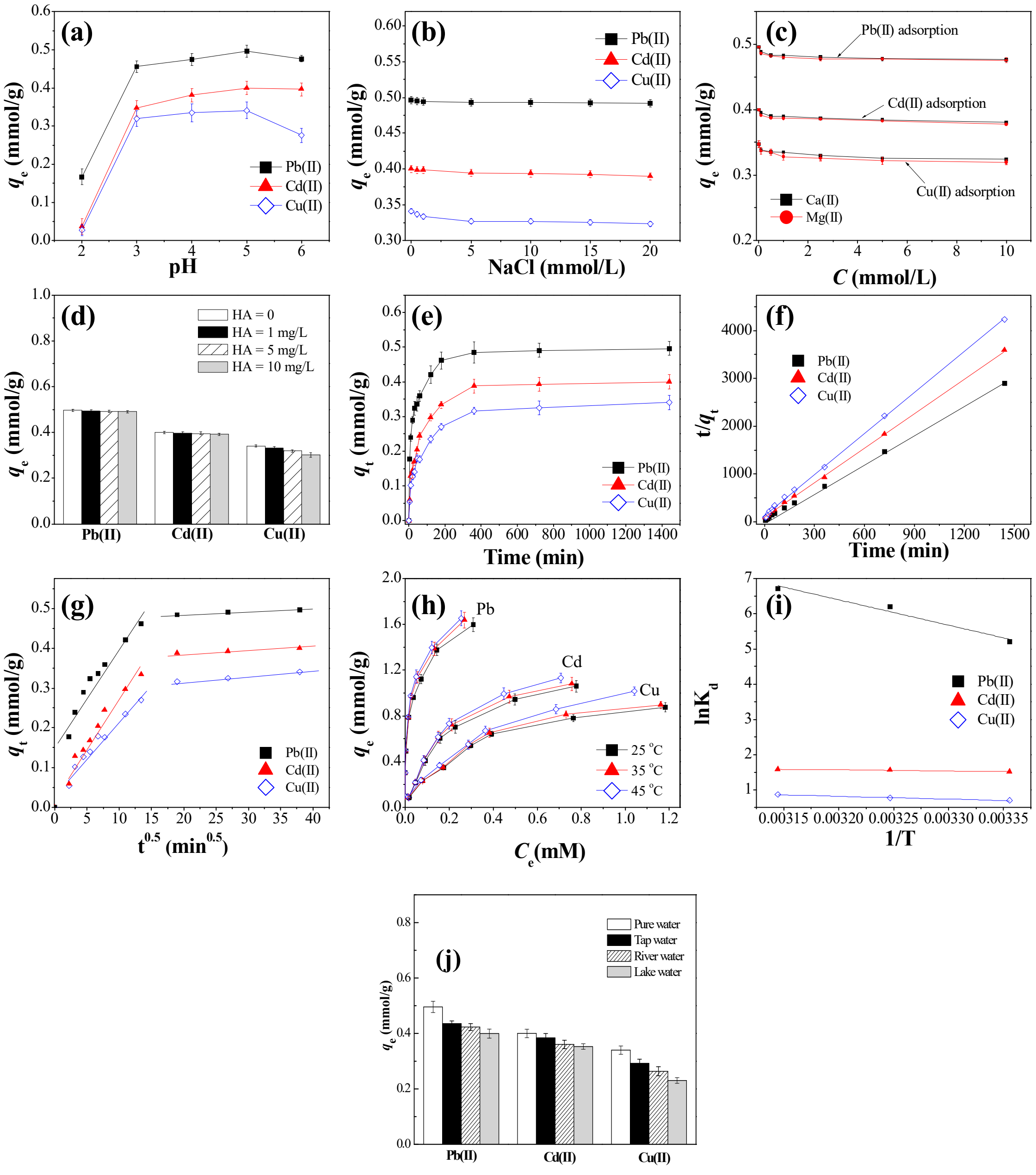

Fig. S5. Effect of different parameters on the adsorption of HMs by EDAR**.** (a) solution pH; (b) ionic strength; (c) Ca(II) and Mg(II); (d) HA. (e) contact time; (f) pseudo-second-order model; (g) intra-particle diffusion model; (h) temperature; (i) plots of ln k_d versus 1/T for the adsorption of HMs by EDAR; (j) effects of different water matrixes. [HMs] = 0.5 mM (except for adsorption isotherm test), [EDAR dosage] = 1.0 g/L, pH 5.0(except for pH test), 25 °C (except for temperature test).

Fig. S6

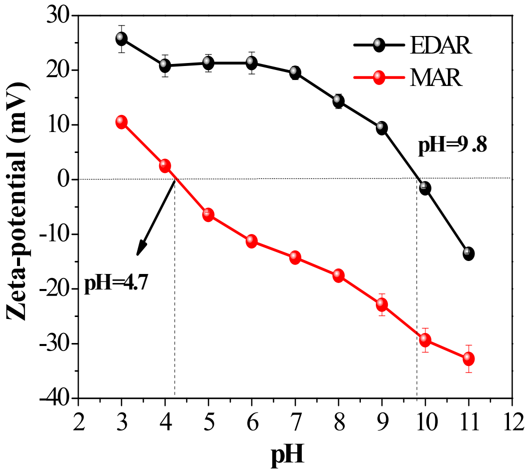

Fig. S6 Variation in zeta potential of EDAR and MAR as a function of pH

Fig. S7

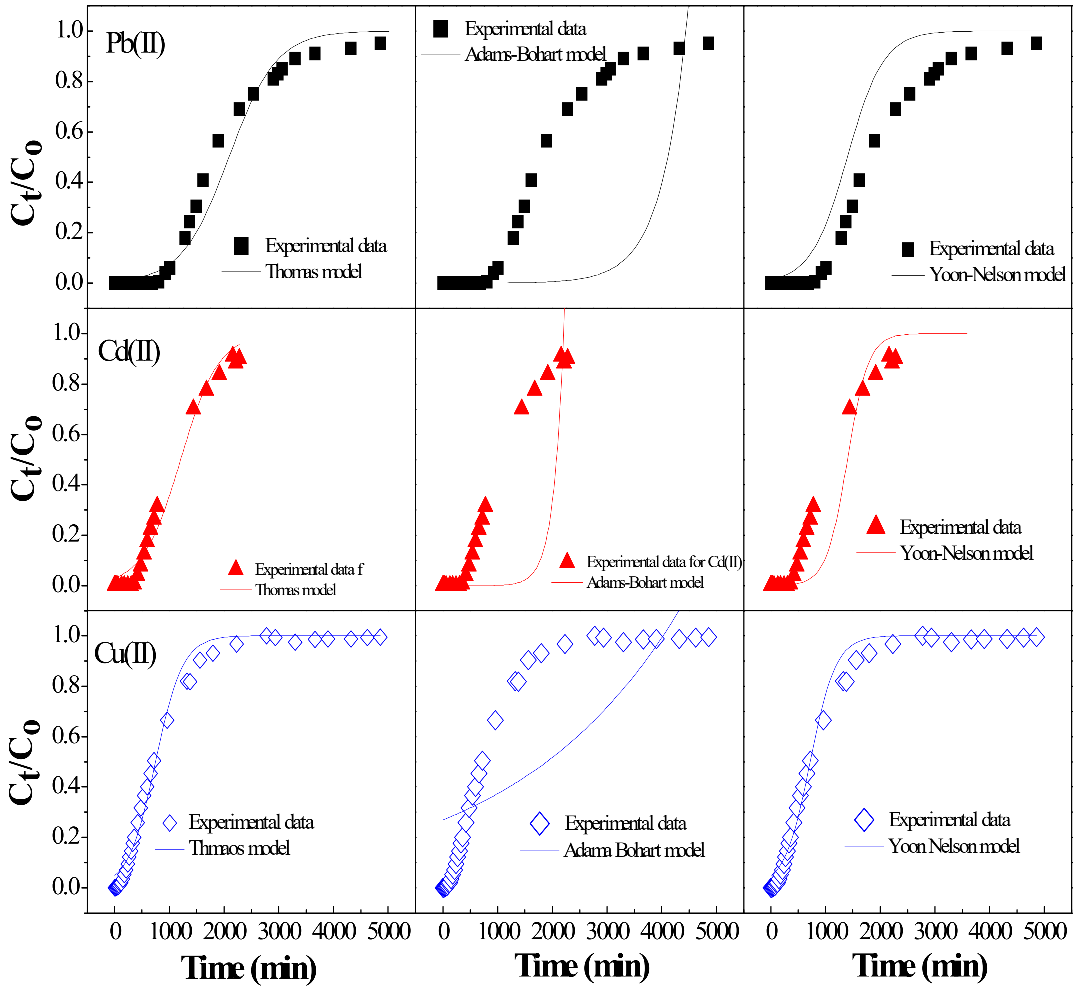

Fig. S7. Comparison of experimental curves for adsorption of Pb(II), Cd(II), and Cu(II) on EDAR with predicted breakthrough curves obtained from the Thomas, Adams Bohart, and Yoon–Nelson models.

Fig. S8

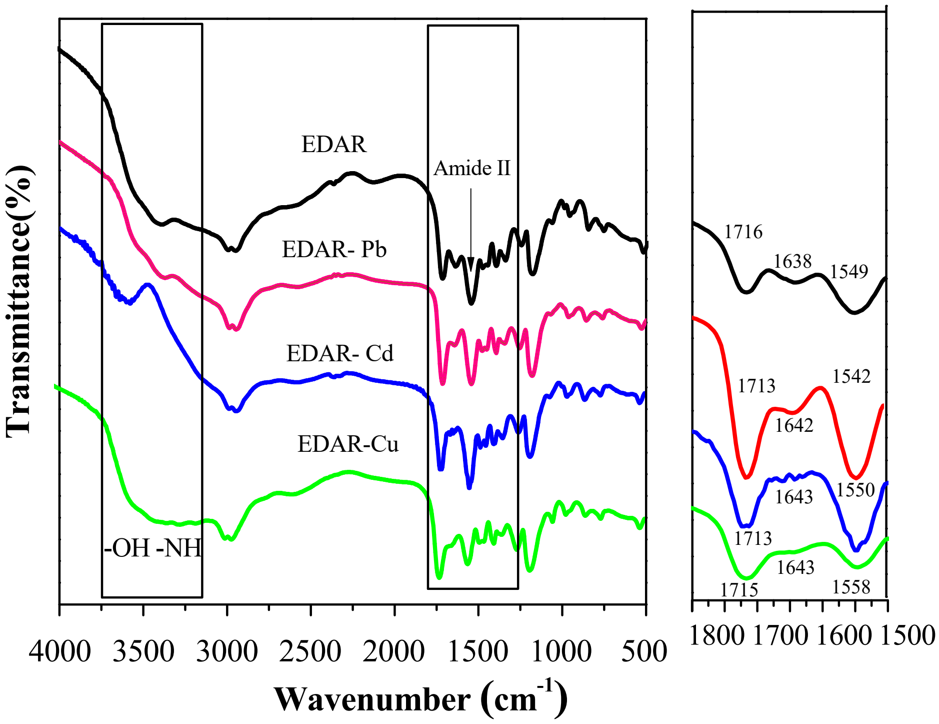

Fig. S8. FTIR spectra of EDAR before and after adsorption of Pb(II), Cd(II), and Cu(II).

Fig. S9

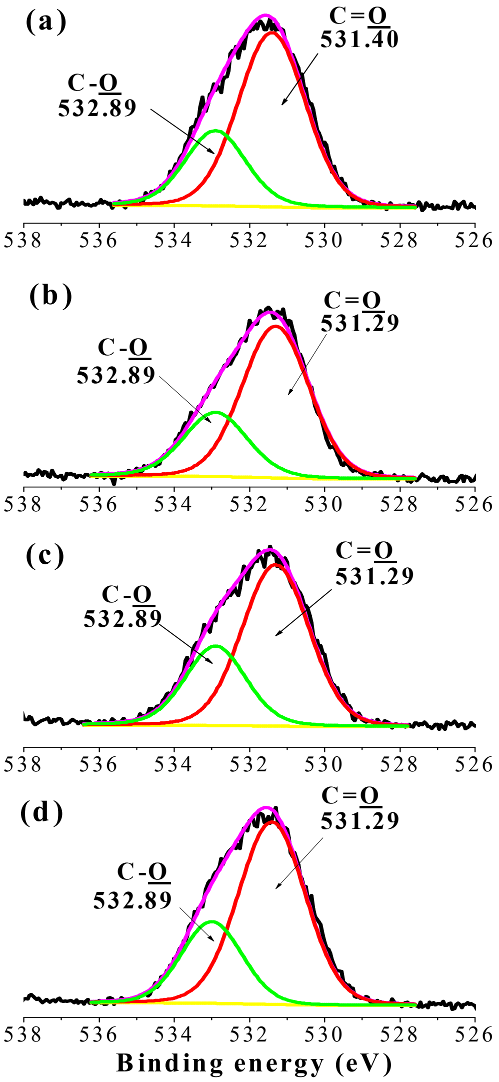

Fig. S9. XPS O1s spectra of EDAR before (a) and after adsorption of Pb(II) (b), Cd(II) (c), and Cu(II) (d).

Fig. S10

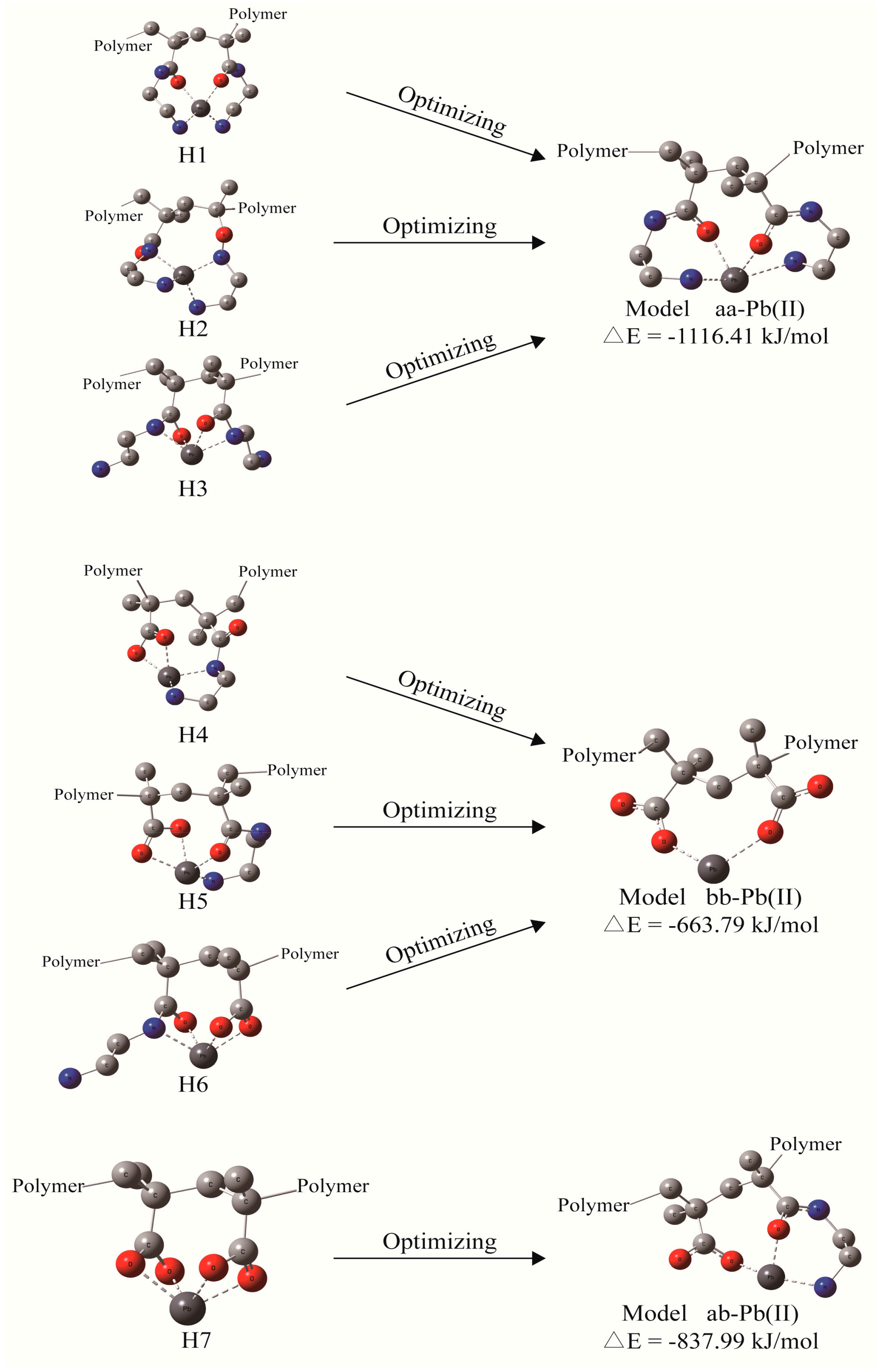

Fig. S10. Initial geometries (H1-H7) used for calculations of Pb(II) coordination to EDAR, and the corresponding optimized coordination geometries (O1-O3).

Table S1

Table S1. Physicochemical properties of commercial adsorbents used in the study.

Adsorbent	Particle size (mm)	BET surface area (m^2/g)	Average pore diameter (nm)	Pore volume (cm^3/g)	Matrix structure	Functional group
Activated carbon		680		0.78		-COOH, -OH
DAX-8	0.24-0.32	75.8	144.2		Polymethylmethacrylate	$-COOCH_3$
D301	0.4−0.7	46.98	274.72	0.18	Polystyrene copolymer	$[-N^+(CH_3)_2]$
D113	0.4−0.7	4.47	37.86	0.52	Polystyrene copolymer	(-COOH)
IRA-410	20-60 mesh				Styrene-divinylbenzene copolymer	$-SO_3H$
IRC 748	0.4−0.7	19.68	16.21	0.079	Styrene-divinylbenzene copolymer	Iminodiacetic acid
L-493		> 1100 [a]	4.6	1.16	Styrene-divinylbenzene copolymer	
XAD-4	0.40-0.70	>750	12.5	0.50	Styrene-divinylbenzene copolymer	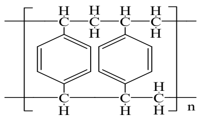

As available and reported by supplier.

Table S2

Table S2. Main characteristics of the natural water samples used in this study

Water	DOC (mg/L)	$SUVA_{254}$ (L/mg·m)	Alkalinity [mM HCO_3^-]	pH	NH_4^+ (mg/L)
Tap water	1.5	1.47	1.0	7.2	0.1
River water	3.4	0.69	1.2	8.0	0.4
Lake water	5.2	0.9	2.0	7.8	0.3

Note: $SUVA_{254}$ (specific ultraviolet absorbance) was calculated from the ultraviolet absorbance at 254 nm (UV_{254}) divided by the DOC.

Table S3

Table S3. Adsorption isotherm model constants for single systems at 25 °C

HMs	Langmuir isotherm			Freundlich isotherm		
	q_{max} (mmol/g)	K_L (L/mmol)	R^2	n	K_F ((mmol/g) $(mmol/L)^{1/n}$)	R^2
Single system						
Pb(II)	1.46	87.61	0.893	0.27	2.25	0.970
Cd(II)	1.34	4.78	0.998	0.47	1.26	0.951
Cu(II)	1.12	3.09	0.991	0.86	0.46	0.959

Table S4

Table S4 Comparison of adsorption capacities of various adsorbents for HMs.

Adsorbent	Adsorbate	Adsorbent capacity (mmol/g)	Isotherm model	pH	T (°C)	Ref.
Polyamine-type starch/GMA copolymer	Pb	1.25	Langmuir	5	25	[11]
	Cd	0.83	Langmuir	5	25	
	Cu	2.33	Langmuir	5	25	
Nanocomposite hydrogels based on wheat bran-g-poly(methacrylic acid) and nano-sized clinoptilolite	Pb	0.81	Langmuir	6.8	25	[12]
	Cd	1.57	Langmuir	6.8	25	
	Cu	3.81	Langmuir	6.8	25	
Thiol-functionalized cellulose nanofiber membranes	Pb	0.15	Langmuir	6	27	[13]
	Cd	0.31	Langmuir	6	27	
	Cu	0.31	Langmuir	6	27	
Lignin-based resin	Pb	0.94	Langmuir	6	25	[14]
	Cd	0.44	Langmuir	6	25	
	Cu	0.94	Langmuir	6	25	
Waste mexerica mandarin “Citrus nobilis” peel	Pb	1.92	Langmuir	5	25	[15]
	Cd	2.88	Langmuir	5	25	
	Cu	2.05	Langmuir	5	25	
Chitosan-iso-vanillin	Cd	0.34	Langmuir	5	30	[16]
Torrefied poplar-biomass	Pb	0.14	Sips	4	20	[17]
Carboxymethylated cellulose fiber	Cu	0.36	Langmuir	6	25	[18]
EDAR	Pb	1.8	Freundlich	5	25	This study
	Cd	1.34	Langmuir	5	25	This study
	Cu	1.12	Langmuir	5	25	This study

Table S5

Table S5. Kinetic parameters for the adsorption of Pb(II), Cd(II), and Cu(II) on EDAR

Model	Parameter	Pb(II)	Cd(II)	Cu(II)
Pseudo-first-order model	$q_{e,\ cal}$ (mmol/g)	0.21	0.20	0.19
	k_1 (1/min)	1.4×10^{-2}	1.3×10^{-2}	9.9×10^{-3}
	R^2	0.873	0.899	0.891
Pseudo-second-order model	$q_{e,cal}$ (mmol/g)	0.50	0.40	0.348
	k_2 (g/mmol/min)	0.12	6.8×10^{-2}	6.7×10^{-2}
	R^2	0.999	0.999	0.999
Intraparticle diffusion model	$k_{id,1}$(mmol/g/min$^{0.5}$)	0.030	0.025	0.020
	$c_{i,1}$	0.11	2.5×10^{-2}	2.4×10^{-2}
	R^2	0.863	0.965	0.962
	$k_{id,2}$(mmol/g/min$^{0.5}$)	6.1×10^{-3}	6×10^{-3}	1.3×10^{-3}
	$c_{i,2}$	0.474	0.375	0.292
	R^2	0.981	0.999	0.996

Table S6

Table S6. Thermodynamic parameters for the adsorption of HMs on EDAR (0.5 mM HMs)

HM	ΔG° (kJ/mol)			ΔH° (kJ/mol)	ΔS° (kJ/mol/K^{-})	R^2
	25°C	35°C	45°C			
Pb(II)	-12.89	-15.90	-17.77	59.9	0.245	0.975
Cd(II)	-3.75	-3.99	-4.19	12.98	0.081	0.987
Cu(II)	-1.73	-1.97	-2.28	6.59	0.028	0.985

Table S7

Table S7. Parameters for the Thomas, Adams–Bohart, and Yoon–Nelson dynamic adsorption models fitted for Pb(II), Cd(II), and Cu(II)

HM	Thomas model			Adams–Bohart model			Yoon–Nelson model	
	q_0 (mmol/g)	K_T (L/min/mmol)	R^2	N_0 (mmol/L)	K_{AB} (ml/mmol/min)	R^2	K_{YN} (1/min)	R^2
Pb(II)	1.63	4.2×10^{-3}	0.982	655.3	1.37×10^{-2}	0.852	2.9×10^{-3}	0.853
Cd(II)	1.20	5.7×10^{-3}	0.979	1245.7	8.17×10^{-3}	0.311	2.0×10^{-3}	0.961
Cu(II)	0.75	2.2×10^{-3}	0.987	1921.5	6.46×10^{-3}	0.602	1.6×10^{-3}	0.856

Table S8

Table S8. Changes in lengths of selected bonds in aa model of EDAR and a result of complexation with Pb(II) (in A°)

Chemical Bonds	aa model (A°)	aa-Pb complex model (A°)	Difference (A°)
C8=O19	1.26	1.30	0.042
C11-N12	1.46	1.50	0.045
C8-N9	1.37	1.34	-0.033
C14=O28	1.26	1.29	0.030
C17-N18	1.47	1.50	0.029
C14-N15	1.36	1.34	-0.02

Supplementary References

[1] Becke, A. D. A new mixing of Hartree–Fock and local density-functional theories. *J. Chem. Phys,* **1993**, 98(2): 1372−1377.

[2] Langmuir I. The adsorption of gases on plane surfaces of glass, mica and platinum. *J. Am. Chem. Soc.* **1918**, 40, 1361−1403.

[3] Freundlich H. Concerning adsorption in solutions. *J. Phys. Chem.* **1906**, 57, 385–470.

[4] Mahamadi, C.; Nharingo, T. Competitive adsorption of Pb^{2+}, Cd^{2+} and Zn^{2+} ions onto Eichhornia crassipes in binary and ternary systems. Biores Technol, 2010, 101, 3: 859–864.

[5] Lagergren S. About the theory of so-called adsorption of soluble substances, *Kungliga Svenska Vetenskapsak ademien Handlingar*. 1898, 24: 1−39.

[6] Ho Y.S.; G. McKay, Pseudo-second order model for sorption processes. *Process. Biochem*. **1999**, 34: 451–465.

[7] Weber, W.J.; Morris, J.C.; Kinetics of adsorption on carbon from solution, J. Sanit. *Eng. Div. Am. Soc. Civ*. Eng. **1963**, 89: 31–60.

[8] Thomas, H. C. Heterogeneous ion exchange in a flowing system. *J Am. Chem. Soc* **1944**, 66(10): 1664–1666.

[9] Yoon, Y. H.; Nelson, J. H. Application of gas adsorption kinetics—II. A theoretical model for respirator cartridge service life and its practical applications. *Am. Ind. Hyg. Assoc. J* **1984**, 45(8): 517–524.

[10] Bohart, G.; Adams, E. Some aspects of the behavior of charcoal with respect to chlorine. 1. *J. Am. Chem. Soc*. **1920**, 42(3): 523–544.

[11] Chen, Y.N.; Zhao, W.; Wang, H.; Meng, X.H.; L.J. Zhang, A novel polyamine-type starch/glycidyl methacrylate copolymer for adsorption of Pb(II), Cu(II), Cd(II) and Cr(III) ions from aqueous solutions. *Roy Soc Open Sci*. **2018**, 5: 180281.

[12] Barati, A.; Moghadam, E.A.; T. Miri, M. Asgari, Rapid removal of heavy metal cations by novel nanocomposite hydrogels based on wheat bran and clinoptilolite: Kinetics, thermodynamics, and isotherms. *Water Air Soil Poll.* **2014**, 225: 2096.

[13] T. Xiang, Z.L. Zhang, H.Q. Liu, Z.Z. Yin, L. Li, X.M. Liu, Characterization of cellulose-based electrospun nanofiber membrane and its adsorptive behaviours using Cu(II), Cd(II), Pb(II) as models. *Sci Chin Chem*, **2013**, 56: 567–575.

[14] Liang, F.B.; Song, Y.L.; Huang, C.P.; Li, Y.X.; Chen, B.H. Synthesis of novel lignin-based ion-exchange resin and its utilization in heavy metals removal. *Ind Eng Chem Res*, 2013, 52: 1267–1274.

[15] Inagaki, C.S.; Caretta, T.D.; Alfaya, R.V.D.; Alfaya, A.A.D.; Mexerica mandarin (Citrus nobilis) peel as a new biosorbent to remove Cu(II), Cd(II), and Pb(II) from industrial effluent. *Desalin Water Treat*, **2013**, 51: 5537–5546.

[16] Alakhras, F. Biosorption of Cd(II) Ions from Aqueous solution using chitosan-iso-vanillin as a low-cost sorbent: Equilibrium, kinetics, and thermodynamic studies. *Arab J Sci Eng*, **2018**, 44: 279–288.

[17] Demey, H.; Melkior, T.; Chatroux, A.; Attar, K.; Thiery, S.; Miller, H.; Grateau, M.; Sastre, A.M.; Marchand, M. Evaluation of torrefied poplar-biomass as a low-cost sorbent for lead and terbium removal from aqueous solutions and energy co-generation. *Chem Eng J*, **2019**, 361: 839–852.

[18] Wang, J.; Liu, M.; Duan, C.; Sun, J.P.; Xu, Y.W. Preparation and characterization of cellulose-based adsorbent and its application in heavy metal ions removal. *Carbohyd Polymer*, **2019**, 206: 837–843.
